# Origin of the High Variability in Sol–Gel Phase Transitions: The Agar Gelation Model

**DOI:** 10.3390/gels12040304

**Published:** 2026-04-02

**Authors:** Claudia Spoliti, Raimondo De Cristofaro, Enrico Di Stasio

**Affiliations:** 1 Department of Translational Medicine and Surgery, Catholic University of the Sacred Heart, Largo Francesco Vito 1, 00168 Rome, Italy; claudia.spoliti@unicatt.it (C.S.); raimondo.decristofaro@unicatt.it (R.D.C.); 2 Foundation University Hospital “A. Gemelli” IRCCS, Largo Agostino Gemelli 8, 00168 Rome, Italy; 3 Department of Basic Biotechnological Sciences, Intensive Care and Perioperative Clinics, Catholic University of the Sacred Heart, Largo Francesco Vito 1, 00168 Rome, Italy

**Keywords:** sol–gel phase transition, gelation variability, gelation heterogeneity, gelation kinetics

## Abstract

Sol–gel phase transitions are complex far-from-equilibrium processes characterized by limited reproducibility, whose origin remains poorly understood and rarely quantified. We investigated the thermally induced sol–gel transition of agar using turbidimetry. A phenomenological model was applied to extract key kinetic parameters (maximum absorbance, maximum rate, and characteristic times) from 96 independent replicates. Variability was quantified and compared with that of an enzymatic reaction exhibiting similar sigmoidal kinetics, allowing for separation of experimental, intrinsic, and nonergodic contributions. Agar gelation displays markedly higher variability. The total variability (CV ≈ 16%) exceeds both the experimental error (1–2%) and the nonergodic contribution (≈2%), demonstrating that it predominantly arises from intrinsic process dynamics. Variability increases sharply during early stages of gelation and then evolves more gradually, indicating that stochastic nucleation and network formation pathways drive divergent kinetic trajectories despite identical initial conditions. Variability in gelation is therefore not a measurement artifact but an intrinsic hallmark of the sol–gel transition. This inherent stochasticity limits the predictive power of deterministic models, particularly at meso- and microscopic scales, and should be considered a fundamental feature of gel-forming systems. Our approach provides a quantitative framework for characterizing variability in phase transitions and may be extended to more complex biological and soft matter systems.

## 1. Introduction

Phase transitions are among the most fundamental phenomena studied in statistical physics [1] and have recently emerged as key mechanisms regulating biomolecular organization and function in biological systems. They govern the assembly of biomolecules into distinct physical states—liquid, gel-like, or solid—thereby influencing biochemical reactions and cellular behavior [2,3,4,5,6]. From a thermodynamic perspective, a phase is defined by an order parameter describing its internal structure, and a phase transition occurs when a control parameter crosses a critical threshold, inducing a categorical change in this order parameter [1,7].

Among these processes, the sol–gel phase transition represents a unique and particularly complex case. In this transition, a liquid colloidal system (sol) evolves into a space-spanning three-dimensional network (gel) through polymerization and cross-linking events, ultimately forming a macroscopically connected structure [8,9,10]. Despite extensive investigation, the sol–gel transition remains only partially understood, mainly due to its pronounced variability and limited reproducibility [1,7,11]. Even small fluctuations in experimental conditions or local heterogeneities can significantly alter the kinetics of aggregation and network formation, leading to distinct structural and mechanical properties in the final gel [9,12,13,14].

Several theoretical models have been proposed to describe the mechanisms underlying gelation, incorporating thermodynamic, kinetic, scale and mechanical aspects of the system [1,7,11,15]. However, when applied to experimental data, these models often fail to fully capture the observed behavior, particularly the large variability associated with the process. A critical but often overlooked aspect is that different experimental techniques probe different spatial and temporal scales. For instance, microscopy methods provide high-resolution structural information but are limited to very small volumes [16,17], whereas light scattering and turbidity measurements enable real-time monitoring of gelation kinetics over larger sample volumes, albeit in a more indirect manner [18,19]. Complementary information can be obtained from calorimetry and rheology, which generally probe macroscopic sample volumes and provide averaged thermodynamic and mechanical properties, while lacking direct information on the microscopic physical structure [20,21]. As a consequence, the observed variability may depend not only on the intrinsic properties of the system but also on the measurement scale and methodology.

Agar is a well-established model system for studying sol–gel transitions due to its simplicity, availability, and well-characterized gelation mechanism. It consists of a mixture of polysaccharides, primarily agarose, which forms thermoreversible gels through the establishment of intra- and intermolecular interactions, leading to a network of rod-like fibrillar structures [22,23,24,25,26]. These features, together with the intrinsic variability inherent to gelation processes, make agar, despite its simplicity, an ideal model system, particularly suitable for the present study, aimed at investigating the fundamental principles governing gel formation.

Despite the widespread observation of variability in gelation processes, its origin is rarely quantified and is often implicitly attributed to experimental uncertainty. In particular, it remains unclear as to what extent variability reflects intrinsic stochastic features of the sol–gel transition rather than external or methodological factors.

In this study, we address this gap by quantitatively investigating the variability of the agar sol–gel transition using time-resolved turbidimetry. By combining large-scale replication with phenomenological modeling of kinetic profiles, we extracted Abs_Max_, V_Max_, t_VMax_, t_AbsMax/2_, and t_AbsMax_ from each experimental curve. We further compared these results to another process based on a completely different biochemical reaction (i.e., the absorbance increase resulting from chromogenic signal generation by enzymatic catalysis), yet producing a very similar absorbance–time profile, namely the S–2238/thrombin assay, used here as a reference assay. This reaction represents a typical enzyme–substrate process with well-defined and reproducible kinetics under controlled conditions, thus providing a suitable benchmark to distinguish intrinsic variability from experimental or methodological contributions. Using this approach, we aim to disentangle the contributions of experimental error, nonergodicity effects and intrinsic process variability. Our results demonstrate that variability is an inherent feature of the sol–gel transition and provide a quantitative framework for its characterization.

## 2. Results and Discussion

Given the primarily methodological nature of this work, readers are encouraged to consult Section 4, located at the end of the manuscript, before reading Section 2.

### 2.1. Kinetic Characterization of the Agar Sol–Gel Transition

**Figure 1:**
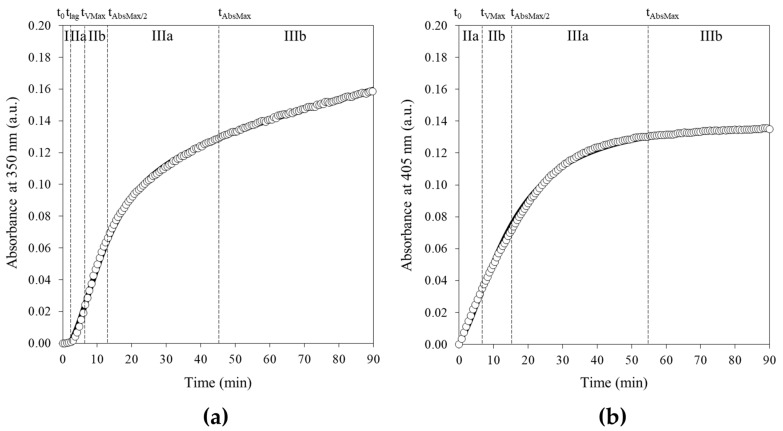
(**a**) Kinetic profiles of the cold-induced reversible gelation of 0.25% agar at 25 °C (absorbance changes at 350 nm plotted vs. time) and their corresponding fitted curves (continuous line, ―). According to Equation (3), the fitting parameters of agar are A = 0.132, B = 12.087, C = 1.731, m = 0.00044, and q = −0.009. Derived kinetical parameters for agar gelation are Abs_Max_ = 0.132, V_Max_ = 0.007 Abs/min, t_VMax_ = 5.9 min, and t_AbsMax/2_ = 11.9 min. Considering the crucial timings t_0_, t_lag_, t_VMax_, t_AbsMax/2_ and t_AbsMax_ (Section 4.4), five phases can be identified: I (lag phase), IIa (increase up to maximum rate), IIb (approximately constant-rate increase), IIIa (decelerating increase), and IIIb (plateau with a slight asymptotic drift); see main text for further details; (**b**) Kinetic profiles of the enzymatic assay of 8 μM S–2238 catalyzed by 0.15 nM human alpha thrombin at 25 °C (absorbance changes at 405 nm plotted vs. time) and their corresponding fitted curves (continuous line, ―). According to Equation (3), the fitting parameters for S–2238 are A = 0.139, B = 15.048, C = 1.742, m = −0.000027, and q = 0.005. Derived kinetical parameters for S–2238 enzymatic assay are Abs_Max_ = 0.136, V_Max_ = 0.006 Abs/min, t_VMax_ = 7.2 min, and t_AbsMax/2_ = 14.9 min. Contrariwise, for the enzymatic reaction only IIa, IIb, IIIa and IIIb phases can be distinguished.

Representative kinetics and phenomenological Equation (3)-fitted curves of the agar thermo-induced gelation and S–2238 enzymatic assay, recorded by the absorbance increase at 350 nm and 405 nm, respectively, are illustrated in Figure 1. The curve fitting analysis allowed for the extraction of key process parameters (Abs_Max_, V_Max_, t_VMax_ and t_AbsMax/2_—Section 4.4), thus providing a quantitative description of the different stages of gelation and forming the basis for subsequent variability analysis and comparation with the reference enzymatic assay.

The 0.25% agar sol–gel transition at 25 °C shows a sigmoidal absorbance profile (Figure 1a). It is possible to identify a multistep process composed of:

- I: a lag phase from the initial time (t_0_) to the onset of the absorbance increase (t_lag_), corresponding to the maximum of the second derivative of absorbance profile with respect to time;
- IIa: a progressive increase in absorbance from t_lag_ up to the maximum rate t_VMax_ (i.e., the maximum of the first derivative), coinciding with the macroscopic appearance of the gel;
- IIb: a gradual absorbance increase proceeding at an approximately constant rate from t_VMax_ to t_AbsMax/2_ (i.e., the minimum of the second derivative);
- IIIa: a slower increase in absorbance from t_AbsMax/2_ to t_AbsMax_;
- IIIb: a final horizontal plateau, often masked by an asymptotic drift (depending on the agar concentration [23]).

Each step was monitored in parallel by macroscopic observation to determine the physical state of the sample (Section 4.3). Together with theoretical models describing the sol–gel phase transition [1,7,11,15] and the gelation mechanism of agar [25,26,27], these observations and results allow for the turbidity kinetic profile to be interpreted in terms of underlying microscopic and macroscopic events, summarized as follows. Referring back to Figure 1a, in the early stage (step I), the sample persists in an apparent sol state for approximately 3 min, with minimal absorbance variation (as directly observed from the data), indicating the absence of significant light-scattering structures. In this regime, monomers are expected to activate and interact, forming low-molecular-weight oligomeric aggregates, as described for agar systems in the literature [25,26,27]. Upon quenching, liquid–liquid phase separation is expected to occur, leading to the formation of polymers nuclei, as reported in previous studies [25,26,27]; however, the clusters remain small and sparsely distributed, producing only a slight increase in the overall turbidity, consistent with the weak absorbance change observed in this stage in Figure 1a. Cooling to the set experimental gelling temperature (25 °C) disrupts the sample consistency and triggers an increase in absorbance in phase IIa, indicating the formation of larger and/or more numerous scattering structures, typically associated with the development of high-molecular-weight polymers in agar gelation [25,26,27]. As gelation progresses, the nuclei grow and assemble into a polymer-rich network, leading to a sharp increase in turbidity that continues until gel formation (phase IIb), as visually confirmed and reported in Section 4.3. This behavior is consistent with established models of sol–gel transition and network formation [1,7,11,15]. Accordingly, the sol–gel phase transition is marked by a sharp rise in absorbance (phases IIa–IIIa), where the slope rapidly increases until reaching an inflection point, after which a plateau is attained (after approximately 45 min under the reported experimental conditions), approaching the maximum absorbance (Abs_Max_), as directly observed in Figure 1a. At this stage (phase IIIb), drift may mask the plateau, giving rise to an apparently oblique asymptote, likely attributed to gravity-induced syneresis and slow structural reorganization within the newly formed three-dimensional space-spanning network, as commonly reported in the literature.

Figure 1b shows a representative kinetic profile of the enzymatic cleavage of S–2238 by thrombin, followed by the release of p-nitroaniline (pNA), which exhibits a characteristic absorbance at 405 nm. The process, based on a completely different biochemical reaction, leads to a similar kinetic profile that can be adequately described by the four parameters obtained from the fitting procedure, thus enabling a direct kinetic comparison between two processes governed by distinct underlying mechanisms.

### 2.2. Large-Scale Variability of the Sol–Gel Transition

To assess reproducibility, large-scale kinetic measurements were performed on agar gelation under identical experimental conditions. Subsequently, to determine whether the variability arises from experimental uncertainty or reflects intrinsic process dynamics, the agar system was compared to the reference enzymatic assay.

**Figure 2:**
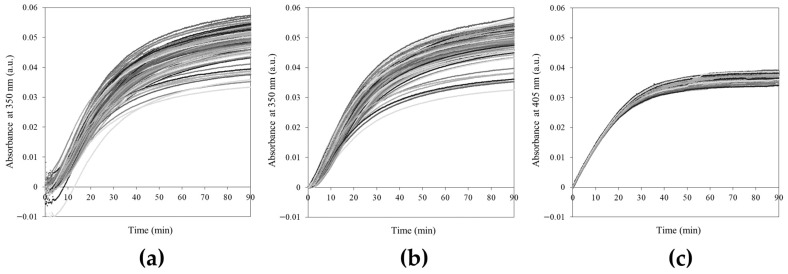
(**a**) Raw kinetic curves of 0.25% agar at 25 °C (absorbance changes at 350 nm); (**b**) Time-trimmed (first 3 min removed) and zero-normalized kinetic curves of 0.25% agar at 25 °C (absorbance changes at 350 nm); (**c**) Kinetic curves of the enzymatic reaction (absorbance changes at 405 nm) between S–2238 and thrombin. All panels: i = 96 (number of measurements).

Figure 2 illustrates the kinetic profiles of 96 samples for both the agar sol–gel phase transition and S–2238 enzymatic assay.

In Figure 2a, raw kinetic curves of 0.25% agar at 25 °C are presented. At early times, the absorbance signal can be unstable as a result of thermal shock induced by the rapid drop of temperature from 75 °C to 25 °C, which requires approximately 3 to 5 min to reach thermal equilibrium (as measured using a thermal probe in 200 μL samples in a 96-well plate and in 2000 μL samples in disposable polystyrene cuvettes, respectively—see Figure S1, Supplementary Materials). Data points collected before thermal equilibrium was reached were discarded and the kinetic curves were normalized to zero (Figure 2b). In order to assess whether the observed high variability of the curves was attributable to the procedure experimental error or to the intrinsic nature of the sol–gel transition, we compared the 96 agar S-shaped curves with those of the S–2238 enzymatic assay, reported in Figure 2c. Experimental conditions were designed appropriately to achieve comparable absorbance profiles and fitted parameters values.

The three key timings t_VMax_, t_AbsMax/2_ and t_AbsMax_ (Section 4.4) represent different stages of the process. The mean values (μ_Abs_) and standard deviations (σ_Abs_) of the corresponding absorbances, together with their relative ratios, are reported in Table 1. At each critical time point, similar absorbance values were obtained in both processes (with agar/S–2238 ratios ranging from 1.46 to 1.13), although completely different reproducibility was observed, as demonstrated by the σ_Abs_ ratios, spanning from 15.15 to 4.00. As shown, the sol–gel transition exhibits a time-dependent absorbance profile, with fluctuations of approximately one order of magnitude compared to the enzymatic reaction signal stability. In fact, at t_VMax_, σ_Abs_ in the agar system is more than 15-fold higher than in the enzymatic reaction. This difference remains substantial at later stages, with σ_Abs_ ratios of ≈10-fold at t_AbsMax/2_ and ≈4-fold at t_AbsMax_. These results demonstrate that the sol–gel transition operates in a fundamentally different reproducibility regime compared to a well-controlled kinetic process.

### 2.3. Temporal Evolution of Variability

**Figure 3:**
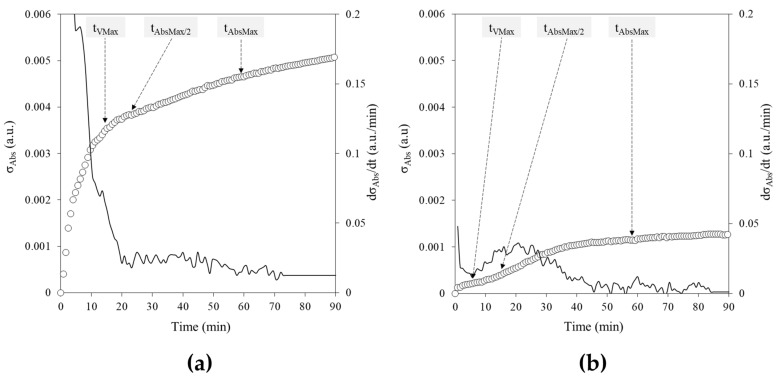
(**a**) Experimental σ_Abs_ of agar gelation process plotted as a function of time, together with its time derivative (dσ_Abs_/dt, continuous line ―) shown on the secondary *Y*-axis; (**b**) Experimental σ_Abs_ of S–2238 enzymatic assay plotted as a function of time, together with its time derivative (dσ_Abs_/dt, continuous line ―) shown on the secondary *Y*-axis. All panels: σ_Abs_ were calculated from different curves (i = 96) at each kinetic time point j of the processes (676 per curve, from 0 to 90 min, acquired at 0.13 min intervals). t_VMax_, t_AbsMax/2_ and t_AbsMax_ are indicated by the arrows for both the sol–gel phase transition and the enzymatic reaction processes: 14.4, 22.2, 58.8 min and 6.7, 15.0, 58.8 min, respectively.

The time-resolved evolution of variability, quantified as σ_Abs_(t)—the ensemble standard deviation calculated across the 96 curves at each time point—and their time derivatives (continuous lines, ―), reveals distinct dynamic patterns for the two systems (Figure 3). In the agar sol–gel transition (Figure 3a), during the early stages of the process, the variability increases sharply, reaching a maximum around t_Vmax_, and it is associated with a steep decrease in dσ_Abs_/dt. As expected, this initial phase is characterized by a rapid amplification of fluctuations, reflected by the sharp increase in σ_Abs_. Beyond this point, variability continues to increase but at a significantly lower rate, as reflected by an approximately constant derivative, then approaching a plateau in the later stages of gelation. In contrast, the enzymatic assay (Figure 3b) exhibits a smoother and more gradual increase in variability, with a bell-shaped profile in its time derivative reflecting a slow–fast–slow rate increase in σ_Abs_. The absence of abrupt changes indicates a more homogeneous and predictable kinetic progression.

These results indicate that the early stages of the sol–gel transition are particularly sensitive to fluctuations, suggesting that variability originates during nucleation and early network formation.

### 2.4. Distribution of Kinetic Parameters

**Figure 4:**
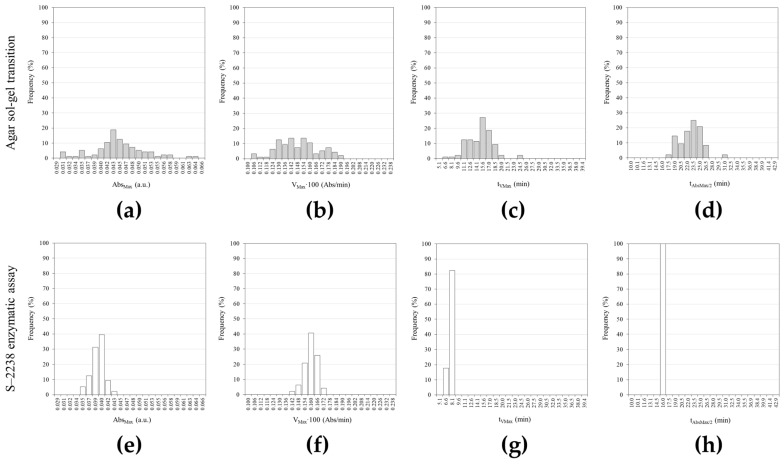
(**a**–**d**) Percentage frequency distributions of Abs_Max_, V_Max_·100, t_VMax_ and t_AbsMax/2_ obtained for the agar sol–gel phase transition; (**e**–**h**) Percentage frequency distributions of Abs_Max_, V_Max_·100, t_VMax_ and t_AbsMax/2_ obtained for the S–2238 enzymatic assay. Statistical comparisons between agar sol–gel transition and S–2238 enzymatic reaction distributions were performed separately for each parameter using a Mann–Whitney U test, indicating highly significant differences in all cases (*p* < 0.0001). All panels: i = 96.

The distributions of the kinetic parameters derived by fitting Equation (3) to the large-scale measurements further highlight the intrinsic variability of the sol–gel phase transition (Figure 4, Table 2).

Figure 4 shows the frequency distributions (%) for the four parameters (Abs_Max_, V_Max_, t_VMax_ and t_AbsMax/2_) describing the time evolution of the agar sol–gel phase transition and S–2238 enzymatic assay. Since the V_Max_ values (Abs/min) were very small, they were multiplied by 100; therefore, they are reported as V_Max_·100.

The main descriptive statistical indices are summarized in Table 2. Abs_Max_ and V_Max_·100 for the two processes exhibit similar mean (μ) values (0.044 vs. 0.038 and 0.145 vs. 0.157, respectively), whereas t_VMax_ and t_AbsMax/2_ (14.4 vs. 6.7 and 22.2 vs. 15.0, respectively) are significantly lower in the enzymatic assay. However, the most prominent feature of the analysis is the noticeably broader spread of each parameter in the sol–gel transition compared to the enzymatic assay. The relative range ratios between the sol–gel and enzymatic processes span from 2.7 for V_Max_·100 to 97.2 for t_AbsMax/2_, thus indicating extreme dispersion in process timing and suggesting intrinsically higher variability of the phase transition phenomenon, independent of the experimental measurement procedure. Similarly, t_VMax_ shows a ≈10-fold broader range, while Abs_Max_ and V_Max_ exhibit ≈4-fold and ≈3-fold increases in dispersion, respectively. These findings demonstrate that variability affects not only the magnitude of the signal but also the temporal organization of the process.

### 2.5. Normalized Variability Profiles

In order to fully compare the absorbance readings obtained from the two kinetics processes, the experimental conditions were selected to yield similar values. As a further step, to make the absorbance data comparable in order to directly evaluate the evolution of variability across the two systems, the mean calculated from the 96 experimental absorbances at each kinetic time point (μ^j^_Abs_—Section 4.7 for notation clarification) was normalized for both processes, resulting in values ranging from 0 to 1, according to the following equation:

(1) $$M_{Abs}^{j}=\frac{\left(\mu_{Abs}^{j}-{Abs}_{Min}\right)}{\left({Abs}_{Max}-{Abs}_{Min}\right)}$$

where μ^j^_Abs_ denotes the mean absorbance at time point j, and M^j^_Abs_ the corresponding normalized mean. Abs_Max_ and Abs_Min_ correspond to the maximum and minimum absorbance values, respectively. The standard deviation of the normalized absorbance (∑^j^_Abs_) was likewise recalculated from σ^j^_Abs_ at each j.

**Figure 5:**
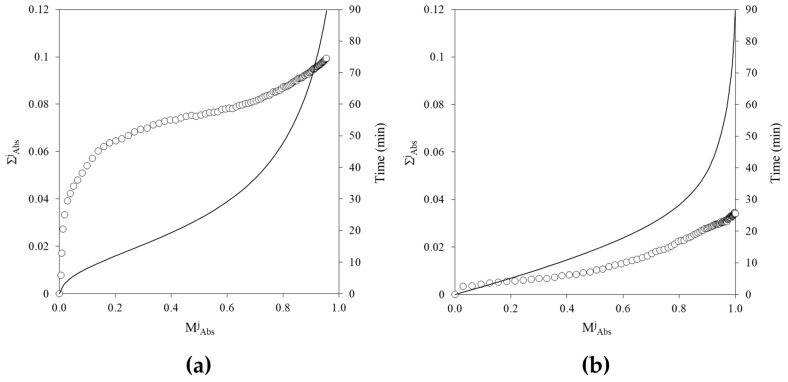
(**a**) Standard deviation of the normalized absorbance at time point j (○ − ∑^j^_Abs_) as a function of the normalized mean absorbance at time point j (M^j^_Abs_) during agar gelation; (**b**) ∑^j^_Abs_ (○) as a function of M^j^_Abs_ during the S–2238 enzymatic assay. All panels: the timing reference is represented by a continuous line (―, secondary *Y*-axis) to provide information on the reaction state associated with each variation of ∑^j^_Abs_. M^j^_Abs_ and ∑^j^_Abs_ were calculated at each time step of the processes across the 96 experiments and normalized according to Equation (1).

The different evolution of the variability associated with the progression of the reactions can be highlighted by plotting ∑^j^_Abs_ as a function of M^j^_Abs_ (Figure 5). To provide a temporal reference for the process phases, time (min) is reported on the secondary *Y*-axis. Completely different variability magnitudes and profiles were observed for the two processes. In Figure 5b, for the enzymatic assay, ∑_Abs_ exhibits a slight linear increase (from 0 to 0.02) as M_Abs_ ranges from 0.0 to 0.75, followed by a steeper increase up to 0.035 at M_Abs_ = 1. In Figure 5a, for the sol–gel phase transition, an exponential growth of ∑_Abs_ is observed during the initial stage, characterized by a low rate of absorbance increase (i.e., as M_Abs_ ranges from 0 to 0.1, a ∑_Abs_ jump from 0 to approximately 0.06 can be detected), followed by a slow linear increase up to 0.08 at M_Abs_ = 0.8, and finally a further increase of about 0.02 points as M_Abs_ spans from 0.8 to its final value.

This divergence indicates that variability in the sol–gel phase transition is amplified early in the process and does not simply scale with the extent of reaction.

### 2.6. The Ergodicity Issue and the Origin of Variability

Nonergodicity arises as the system evolves from a sol to a gel state during the phase transition [28,29]. In the solution phase, concentration fluctuations pervade the entire space uniformly, thereby making time and ensemble averages identical (ergodic system), as the system evolves through all representative fractions of the configurational space given sufficient time. Upon gelation, the system undergoes an ergodic-to-nonergodic transition, and the previously described condition no longer exists because fluctuations become constrained and the spatial configurations explored no longer span the entire configurational space [28,29,30].

From an experimental point of view, this phenomenon can be proven by comparing the values of an observable—absorbance, in our case (Section 4.6). Absorbance can be obtained either as a “time average”, using a sequential time recorder at a single spatial point, or as an “ensemble average” derived from multiple measurements taken at different spatial points of the system. Both averages are dependent on the physical scale of the observables (time and space). When the observed volume is sufficiently large, microscopic fluctuations—characteristic of a nonergodic, restrictive scenario—can average out over multiple ensembles, causing the system to appear ergodic at the macroscopic scale. In our experimental set-up, the controlled volume ranged from approximately 5 to 15 mm^3^, depending on the dimension and optical path length of the light beam of the microplate reader or conventional spectrophotometer as it passes through the sample (≈1 s as integration time). Data are reported in Table 3.

To quantify the different contributions, the total variability (Var_tot_) of the experimentally recorded absorbance can be expressed as the sum of different contributions, according to the following equation:

(2) $${Var}_{tot}={Var}_{exp}+{Var}_{prc}+{Var}_{n-erg}$$

where Var_exp_ is the contribute of the experimental procedure (experimental error), Var_prc_ is the intrinsic variability of the observed phenomenon, related to the dimensionality of the process (i.e., the number of variables involved) or to the presence of chaotic behavior, and Var_n-erg_ is the signal fluctuation derived from the nonergodic scenario.

For our systems, Var_tot_, in terms of the coefficient of variation (CV) calculated from the statistical indices of Abs_Max_ reported in Table 2, is 15.9% and 5.3% for the agar gel and the enzymatic assay, respectively. Var_exp_, arising from user-induced and experimental errors (e.g., dilution and spectrophotometric measurements), is obviously the same and estimated to be on the order of 1–2%. Moreover, as described in Section 4.6, Var_n-erg_ was experimentally measured (Table 3) from readings taken at different cuvette positions and is very close to that of the reference enzymatic assay (2.0% vs. 1.6%, respectively). The latter is ergodic by definition, since it consists of a homogeneous solution, indicating that the observed variability in the sol–gel transition is also independent of any potential nonergodic scenario. Therefore, Var_prc_, the intrinsic variability of the process, is mainly responsible for the huge difference in the resulting overall variability in the sol–gel phase transition. In particular, the magnitude of variability cannot be explained by measurement uncertainty or sampling effects but reflects the stochastic nature of gelation.

### 2.7. Overall Discussion

The central finding of this study is that the large variability observed in the agar sol–gel transition is intrinsic to the process and cannot be accounted for by experimental error or nonergodic effects. Quantitative analysis demonstrates that variability in gelation kinetics exceeds experimental noise by an order of magnitude and remains significantly higher than that observed in a reference enzymatic assay, despite comparable macroscopic kinetic profiles. Overall, these findings establishes variability as a fundamental property of the sol–gel transition rather than a limitation of measurement.

The origin of this intrinsic variability can be attributed to the stochastic nature of the microscopic events underlying gelation. The sol–gel transition involves nucleation, growth, and interconnection of polymer clusters, processes that are inherently sensitive to inhomogeneities, diffusion effects, local fluctuations in concentration, interaction strength, and spatial organization [31,32,33]. As a result, multiple microscopic pathways can lead to the formation of a macroscopically similar gel [9,11,15]. These distinct pathways generate divergent kinetic trajectories, even under identical initial conditions, giving rise to the broad dispersion observed in both temporal parameters and absorbance profiles.

The temporal evolution of variability provides further insight into the mechanism. The sharp increase in variability during the early stages of the process, up to t_VMax_, suggests that fluctuations are amplified during nucleation and early cluster growth. In this regime, small differences in local configurations can propagate and be magnified as the network develops. Beyond this point, variability continues to increase but at a slower rate, consistent with a system that has already established a percolating structure and undergoes more constrained reorganization. This behavior indicates that the early stages of gelation play a dominant role in determining the overall variability of the process.

A key implication of these findings is that sol–gel transitions cannot be fully described within a purely deterministic framework. While macroscopic observables such as final gel formation may appear reproducible, the underlying pathways leading to these outcomes are inherently probabilistic. Consequently, predictive models based solely on average behavior may fail to capture the full range of possible system dynamics, particularly at mesoscopic and microscopic scales. Moreover, depending on the experimental methods used to investigate the phenomenon or on the physical theory describing its mechanical evolution, different system volumes may be probed and different micro- or macroscopic properties may be monitored. As a result, the observed or theoretically predicted variability refers to different scales of the system [34]. In this context, variability should be regarded as an essential observable, providing information about the distribution of accessible states rather than as noise to be minimized.

The comparison with the enzymatic reaction is particularly informative in this regard. Despite producing similar sigmoidal kinetic profiles, the reference enzymatic assay exhibits low variability and a smooth temporal evolution, consistent with a homogeneous and well-defined reaction pathway. In contrast, the sol–gel transition displays large fluctuations and broad parameter distributions, reflecting the multiplicity of underlying microscopic trajectories. Therefore, the generalization of models describing phase transitions is challenging, as the underlying physical processes cannot be adequately captured by statistical mean governing parameters alone, but instead depend strongly on their broad distributions. This contrast highlights a fundamental distinction between systems governed by well-defined molecular mechanisms and those emerging from collective, self-organizing processes.

## 3. Conclusions

From a broader perspective, the behavior observed in agar gelation is expected to extend to a wide class of soft matter and biological systems undergoing phase transitions. Protein condensation, fibrin clot formation, and intracellular phase separation processes all involve similar mechanisms of nucleation, growth, and network formation. In such systems, intrinsic variability may also play a critical functional role, potentially contributing to structural diversity and adaptability. Although this interpretation remains to be further validated, it is consistent with emerging views on the role of stochasticity in biological organization. The framework presented here provides a quantitative approach to characterize this variability and may be applied to more complex and biologically relevant systems.

This study has some limitations. The analysis is restricted to a single model system (agar) under specific experimental conditions, and further work is needed to assess the generality of the observed behavior across different materials, concentrations, and environmental parameters. In addition, the phenomenological model employed here provides a robust descriptive framework but does not explicitly account for the underlying molecular mechanisms. Future studies integrating structural characterization techniques and theoretical modeling could provide deeper insight into the link between microscopic dynamics and macroscopic variability.

In conclusion, the sol–gel transition should be viewed as an intrinsically stochastic process in which variability is a defining feature rather than an experimental artifact. Recognizing and quantifying this variability is essential for a comprehensive understanding of gelation phenomena and for the development of predictive models capable of capturing the full complexity of phase transitions in soft and biological matter.

## 4. Materials and Methods

### 4.1. Materials

Agar (C_12_H_18_O_9_)_x_ powder, having a relative molecular mass (M_r_) of 3000–9000 and a gel point ≈35 °C (for 1.5% solution), as specified by the manufacturer, was purchased by Sigma-Aldrich (St. Louis, MO, USA). The chromogenic substrate for thrombin S–2238, whose chemical name is H-D-Phenylalanyl-L-pipecolyl-L-arginine-p-nitroaniline dihydrochloride (H-D-Phe-Pip-Arg-pNA · 2 HCl), was purchased by Chromogenix (Werfen, Barcelona, Spain). Human alpha thrombin was purchased by Enzyme Research Laboratories (South Bend, IN, USA). PEG 8000, HEPES and sodium chloride were purchased by Sigma-Aldrich (St. Louis, MO, USA).

### 4.2. Agar Gel Preparation and Choice of the Optimal Experimental Configuration

Agar powder was weighed and suspended directly in 30 mL of cold ultrapure water (resistivity 18.2 MΩ·cm at 25 °C) in a 50 mL beaker covered with aluminum foil to obtain different concentrations: 0.1, 0.25, 0.5, 0.75, 1.0% *w*/*v*. After approximately 10 min of heating under continuous magnetic stirring (with a small PTFE-coated stir bar) on a magnetic hot plate set to 100 °C, the boiling point of water was reached and agar was completely dissolved (sol state). Stirring was maintained continuously thereafter, and the hot plate was then set to 75 °C to allow for controlled cooling. The agar solution was kept at this temperature under stirring until the beginning of the experiments. This condition was chosen to maintain the sol state and prevent premature gelation prior to the experiments. The beaker remained covered with aluminum foil throughout the entire process (heating, cooling, and temperature maintenance) to minimize evaporation.

For the evaluation of the optimal conditions of temperature, concentration and recording time, all turbidimetric kinetic profiles were performed at a wavelength of 350 nm to optimize the signal-to-noise ratio, as the intensity of light scattering for rod-like particles increases with the inverse of the fourth power of the wavelength [35,36,37], using a Cary 60 UV–Vis spectrophotometer (Agilent, Santa Clara, CA, USA) equipped with a water-thermostatted 18-cell holder connected to a circulating water bath providing temperature control.

The freshly prepared hot agar solutions, kept at 75 °C under magnetic stirring, were pipetted (2 mL) into disposable polystyrene cuvettes (Sigma-Aldrich, St. Louis, MO, USA) inserted into the spectrophotometer set at the desired experimental temperature to induce the thermo-assisted sol–gel phase transition. A temperature range between 15 and 35 °C (15, 20, 25, 30, and 35 °C) was initially explored to identify optimal conditions. The final selected experimental setup was 0.25% agar at 25 °C, monitored for 90 min.

### 4.3. Macroscopic Events of Agar Sol–Gel Transition

Simultaneously with the turbidity measurements, several samples prepared in parallel under identical experimental conditions were visually inspected, in order to associate the turbidimetric kinetic profile (Figure 1a) with visible physical phenomena, and some key macroscopic observations of the agar sol–gel transition attesting to the occurrence of the process were:

1. An increase in viscosity (the liquid gradually thickens, becoming syrup-like), followed by a loss of fluidity until gelation occurs (the liquid loses its ability to flow and becomes an elastic solid);
2. An increase in the concavity of the solution meniscus as the transition evolves (i.e., the curved surface of the liquid in the cuvette);
3. The gel structure formation, which occurs through the development of a system-spanning network of particles or polymers, creating a continuous solid phase accompanied by increased sample opalescence (turbidity) and the emergence of characteristics elastic properties.

The timing of these macroscopic events was recorded by the three authors with a temporal resolution on the order of minutes.

### 4.4. Fitting Curve Studies

Kinetic curves of the sol–gel transition were measured as an increase in absorbance at 350 nm as a function of time and, consequentially, were fitted using the following phenomenological equation, proposed here to describe the time-dependent evolution of the absorbance signal:

(3) $$Absorbance=\frac{A}{\left[1+\left(\frac{B}{t}\right)c\right]}+\left(m\cdot t\right)+q$$

where A, B, c, m and q are fitting parameters and t is the process time (min).

From the analytical solution of Equation (3), different parameters describing the evolution of the process over time, as indicated and reported in Figure 1a, were derived:

1. Abs_Max_, i.e., the maximum asymptotic absorbance value (corrected by the drift effect);
2. V_Max_, i.e., the maximum slope (dA/dt) of the gelation curve determining the rate of the absorbance increase (Abs/min), hence indicating the rate of the gelation [37,38];
3. t_VMax_, i.e., the corresponding time (min) of V_Max_;
4. t_AbsMax/2_, i.e., the time needed to reach the half of the maximum absorbance value, in turn expression of the entire gelation process rate (min);
5. t_AbsMax_, i.e., the time at which 99% of the asymptotic Abs_Max_ was reached.

### 4.5. A Large-Scale Comparison Between Sol–Gel Phase Transitions and Enzymatic Catalysis Processes

Our preliminary observations had shown a relevant difference among agar sol–gel kinetic curves under the same trial set-up. Therefore, analogous turbidity kinetics on a large number of samples were recorded under similar experimental conditions using a 96-well flat-bottom polystyrene microplate (Corning, Glendale, AZ, USA) on a SpectraMax Abs Plus microplate reader (Molecular Devices, San Jose, CA, USA), operated under temperature-controlled conditions, to study the reproducibility of the sol–gel phase transition event. Samples (200 μL/well) were added using an electronic multichannel pipette to minimize the time between sample addiction and absorbance acquisition (approximately 10 s), thereby ensuring reproducible and identical initial conditions across wells. Absorbance readings were started immediately and were recorded at 25 °C for 90 min, with 676 time points acquired at 0.13 min intervals (i.e., 8 s), each corresponding to a full microplate read, yielding 676 data points per well. Data acquisition was performed column-wise, with each column read in less than 1 s, ensuring minimal temporal variability across wells and avoiding systematic positional effects between edge and central regions of the plate. To assess whether the variability of the results (Figure 2a,b) was attributable to the experimental error associated with the procedure, the same experimental approach was applied to another process, based on a completely different biochemical reaction (i.e., the absorbance increase resulted from chromogenic signal generation by enzymatic catalysis), but producing a very similar absorbance–time profile.

Briefly, in a 96-well flat-bottom polystyrene microplate (final volume 200 μL/well), S–2238 (final concentration 8 μM) was added to human alpha thrombin (final concentration 0.15 nM) in a 0.15 M NaCl, 0.1% PEG 8000, 10 mM HEPES buffer at pH 7.4. The release of pNA was followed at 405 nm [35,39]. Absorbance readings were started immediately after sample mixing and were recorded at 25 °C for 90 min, following the same sample loading and data acquisition procedure described above. Finally, Equation (3) was used to fit the resulting 192 experimental kinetic curves to obtain the corresponding Abs_Max_, V_Max_, t_VMax_, t_AbsMax/2_ and t_AbsMax_. All recorded kinetic curves were included in the analysis and no data were excluded based on post hoc criteria. For each parameter, statistical descriptive analysis and frequency distribution curves were derived and compared. Statistical comparisons between agar sol–gel transition and S–2238 enzymatic reaction distributions were conducted separately for each parameter using a Mann–Whitney U test.

### 4.6. Ergodicity Analysis via Time–Ensemble Average Comparison

To assess the variability arising from the potentially nonergodic gel medium, absorbance measurements were performed on both agar gel and S–2238/thrombin solution samples, each contained in a single four-sided clear disposable fluorescence cuvette (Sigma-Aldrich, St. Louis, MO, USA), using the Cary 60 UV–Vis spectrophotometer. After 2 h of incubation at 25 °C, once the enzymatic reaction had taken place and the agar sample was fully gelled, 100 absorbance readings were collected from each cuvette at a fixed spatial point (integration time 1 s; approximately 2 s between successive readings). In addition, using the same cuvettes, 100 additional absorbance readings were obtained from different spatial points by vertically moving and rotating the cuvette between measurements (integration time 1 s, approximately 2 s between successive readings).

### 4.7. Conventions for Figures and Tables

Unless otherwise stated, the following notation is used in all plots and tables throughout the manuscript:

- i: curve (well) index, 1–96;
- j: time point of the kinetic measurement, 0–90 min (676 points, acquired at 0.13 min intervals);
- t_j_: specific time (e.g., t_VMax_ denotes the time at which V_Max_ occurs);
- Abs_j_: absorbance recorded at specific time (e.g., Abs_Max_ denotes the maximum value of absorbance at t_j_ 90 min).

## Figures and Tables

**Table 1:** Mean absorbance values (μ_Abs_) and corresponding standard deviations (σ_Abs_) of both agar sol–gel phase transition and S–2238 enzymatic assay curves measured at three relevant time points of the processes (t_VMax_, t_AbsMax/2_ and t_AbsMax_). The sol–gel phase transition/enzymatic assay ratios (agar/S–2238) calculated for μ_Abs_ and σ_Abs_ are also reported.

Relevant Time Points	Agar Sol–Gel Transition (i = 96)		S–2238 Enzymatic Assay (i = 96)		Agar/S–2238 Ratios	
t_j_ (min)	μ_Abs_ (a.u.)	σ_Abs_ (a.u.)	μ_Abs_ (a.u.)	σ_Abs_ (a.u.)	μ_Abs_ (a.u.)	σ_Abs_ (a.u.)
**t_VMax_**	0.0137	0.0035	0.0094	0.0002	1.46	15.15
**t_AbsMax/2_**	0.0239	0.0038	0.0194	0.0004	1.23	9.55
**t_AbsMax_**	0.0440	0.0046	0.0388	0.0012	1.13	4.00

t_j_ = specific time; a.u. = arbitrary units; i = number of measurements.

**Table 2:** The main descriptive statistical indices for the four parameters (Abs_Max_, V_Max_ as V_Max_·100, t_VMax_ and t_AbsMax/2_) obtained from the curve-fitting analysis of the agar sol–gel transition and the S–2238 enzymatic assay: mean (μ), standard deviation (σ), coefficient of variation (CV, %), median (Median) minimum (Min), maximum (Max) and range (Range) values. The sol–gel phase transition/enzymatic assay (agar/S–2238) Range ratios are also reported.

	Agar Sol–Gel Transition (i = 96)				S–2238 Enzymatic Assay (i = 96)			
Descriptive Statistics	Abs_Max_ (a.u.)	V_Max_·100 (Abs/min)	t_VMax_ (min)	t_AbsMax/2_ (min)	Abs_Max_ (a.u.)	V_Max_·100 (Abs/min)	t_VMax_ (min)	t_AbsMax/2_ (min)
**μ**	0.044	0.145	14.386	22.200	0.038	0.157	6.736	15.043
**σ**	0.007	0.020	3.101	2.708	0.002	0.006	0.716	0.051
**CV (%)**	15.9	13.8	21.6	12.2	5.3	3.8	10.6	0.3
**Median**	0.043	0.146	14.800	22.933	0.039	0.157	7.067	15.067
**Min**	0.030	0.101	6.133	16.933	0.034	0.139	5.200	14.933
**Max**	0.063	0.186	23.867	29.867	0.043	0.171	7.067	15.067
**Range**	0.033	0.085	17.733	12.933	0.008	0.032	1.867	0.133
**Range ratios** **(agar/S–2238)**	4.1	2.7	9.5	97.2				

a.u. = arbitrary units; i = number of measurements.

**Table 3:** Comparison of absorbance data obtained from temporal and spatial ensemble averages of measurements performed on agar gel and S–2238/thrombin solution after 2 h of incubation at 25 °C.

Absorbance Measurements (i = 100)	Agar Sol–Gel Transition (Mean ± SD, CV%)	S–2238 Enzymatic Assay (Mean ± SD, CV%)
**Time-Averaged** *fixed cuvette position*	0.187 ± 0.002 (0.8)	0.199 ± 0.001 (0.5)
**Spatially Averaged** *variable cuvette positions*	0.192 ± 0.004 (2.0)	0.201 ± 0.003 (1.6)

Values are expressed as mean ± standard deviation (SD); CV = coefficient of variation; i = number of measurements.

## Data Availability

The raw data supporting the conclusions of this article will be made available by the authors on request.

## Supplementary Materials

The following supporting information can be downloaded at https://www.mdpi.com/article/10.3390/gels12040304/s1. Figure S1. Temperature equilibration profiles during the sol–gel transition of 0.25% agar (thermal probe, 10 s resolution; n = 10) for samples subjected to cooling from 75 °C to 25 °C. Black profile corresponds to 2000 μL samples in cuvettes (see Section 4.2), while white profile corresponds to 200 μL samples in 96-well plates (see Section 4.5). Data points are reported as mean ± standard deviation. The data show equilibration within 5 min in cuvette samples and 3 min in 96-well samples.

## Abbreviations

The following abbreviations are used in this manuscript:

CV	Coefficient of Variation
Abs_Max_	maximum asymptotic absorbance (drift-corrected)
V_Max_	maximum rate of the absorbance–time curve
t_VMax_	time at which V_Max_ occurs
t_AbsMax/2_	time required to reach half of Abs_Max_
t_AbsMax_	time at which 99% of Abs_Max_ is reached
pNA	p-nitroaniline
Var_tot_	total variability
Var_exp_	experimental error
Var_prc_	intrinsic variability of the process
Var_n-erg_	nonergodic contribution to the process
