# Supplementary material for: Origin of the High Variability in Sol–Gel Phase Transitions: The Agar Gelation Model
Source: Gels. 2026 Apr 2;12(4):304. doi: 10.3390/gels12040304 (PMC13116384; doi:10.3390/gels12040304)

# SUPPLEMENTARY MATERIALS

for: "Origin of the High Variability in Sol–Gel Phase Transitions: The Agar Gelation Model"

*Claudia Spoliti, Raimondo De Cristofaro and Enrico Di Stasio*

**Figure S1.** Temperature equilibration profiles during the sol–gel transition of 0.25% agar (thermal probe, 10 s resolution; n = 10) for samples subjected to cooling from 75 °C to 25 °C. Black profile corresponds to 2000 µL samples in cuvettes (see Section 4.2), while white profile corresponds to 200 µL samples in 96-well plates (see Section 4.5). Data points are reported as mean ± standard deviation. The data show equilibration within 5 min in cuvette samples and 3 min in 96-well samples.

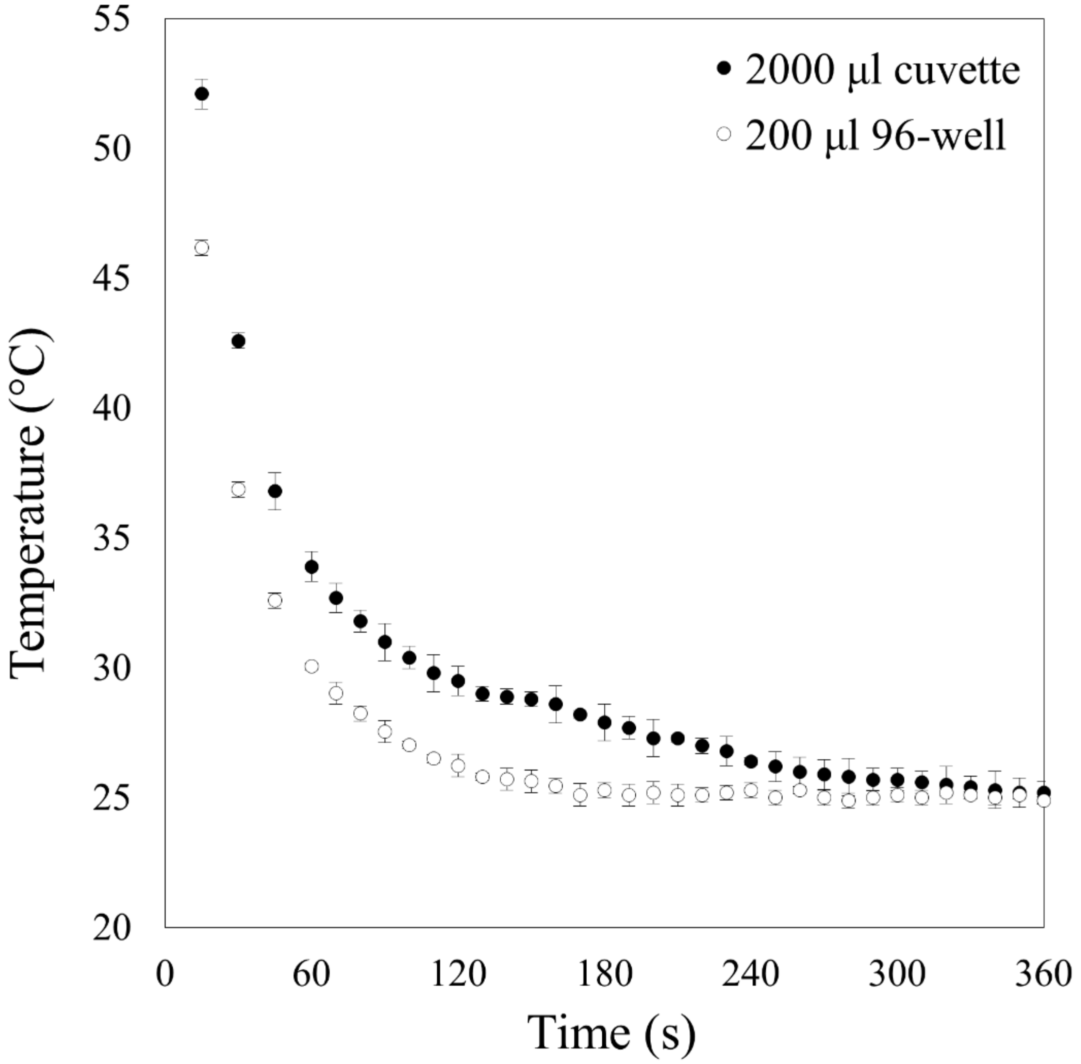

Supplement: Supplementary file 1 [file gels-12-00304-s001.zip › gels-4225755-supplementary.pdf]
